# A Compend of Anatomy and Physiology

**Published:** 1891-09

**Authors:** 


A COMPEND OF ANATOMY AND PHYSIOLOGY. Illustrated by the New Model Anatomical Manikin, including a Key, a Glossary of Medical Terms, and Incidental Notes of Pathology. Edited and compiled from standard works by M. C. Tiers. New York, Fowler & Wells Co., 775 Broadway, 1891.

This small volume is, as stated in its title-page, a literal transcript of which appears above, designed to explain the model anatomical manikin, the advertisement of which appears on the third page of the cover of this journal. With that to illustrate it, the book is an excellent ready reference book for refreshing the memory upon doubtful points of anatomy.

As for the manikin, too much cannot be said in praise of it. A copy of it graces our office, and we find continual occasion to refer to it, and we have never been disappointed in our search for information. The book is, however, a necessary adjunct. W. M. J.

